# Real-world safety and effectiveness of radium-223 in Japanese patients with castration-resistant prostate cancer (CRPC) and bone metastasis: exploratory analysis, based on the results of post-marketing surveillance, according to prior chemotherapy status and in patients without concomitant use of second-generation androgen-receptor axis-targeted agents

**DOI:** 10.1007/s10147-020-01850-3

**Published:** 2021-02-11

**Authors:** Hirotsugu Uemura, Naoya Masumori, Shunji Takahashi, Makoto Hosono, Seigo Kinuya, Toshiyuki Sunaya, Tomoyo Horio, Yutaka Okayama, Yoshiyuki Kakehi

**Affiliations:** 1 Department of Urology, Faculty of Medicine, Kindai University, 337-2, Ono-higashi, Osaka Sayama-City, Osaka, Japan; 2 Department of Urology, Sapporo Medical University School of Medicine, 291, Minami 1-jo Nishi 16-chome, Chuo-ku, Sapporo, Hokkaido Japan; 3 Department of Medical Oncology, The Cancer Institute Hospital of JFCR, 3-8-31Koto-ku, AriakeTokyo, Japan; 4 Department of Radiology, Faculty of Medicine, Kindai University, 337-2, Ono-higashi, Osaka Sayama-City, Osaka, Japan; 5 Department of Nuclear Medicine, Institute of Medical, Pharmaceutical and Health Sciences, Kanazawa University, 13-1 Takaramachi, Kanazawa, Japan; 6 Data Sciences & Analytics, Research & Development Japan, Bayer Yakuhin, Ltd., 2-4-9 Kita-ku, Umeda, Osaka, Japan; 7 Medical Affairs Oncology, Medical Affairs & Pharmacovigilance, Bayer Yakuhin, Ltd., 2-4-9 Kita-ku, Umeda, Osaka, Japan; 8 Pharmacovigilance Monitoring & Governance PMS, Medical Affairs & Pharmacovigilance, Bayer Yakuhin, Ltd, 2-4-9 Kita-ku, Umeda, Osaka, Japan; 9 Department of Urology, Faculty of Medicine, Kagawa University, 1750-1 Ikenobe, Miki-cho, Kita-gun, Kagawa, Japan

**Keywords:** Japanese patients, Bone metastases, Castration-resistant prostate cancer, Post-marketing surveillance, Radium-223, Real-world data

## Abstract

**Background:**

Based on results from Japanese post-marketing surveillance, exploratory analyses were performed to investigate real-world outcomes of radium-223 for metastatic CRPC (mCRPC) according to patient characteristics.

**Methods:**

This non-interventional, prospective study enrolled mCRPC patients selected for radium-223 treatment in clinical practice. Six-month safety and effectiveness were evaluated in subgroups who had/had not received prior chemotherapy (prior-chemo/no prior-chemo groups), and a subgroup who had not received concomitant androgen-receptor axis-targeted agents (ARATs).

**Results:**

In the overall population (*n* = 296), the prior-chemo group (*n* = 126) tended to have more bone metastases, more analgesic use, and higher prostate-specific antigen values than the no prior-chemo group (*n* = 170). Incidences of treatment-emergent adverse events (TEAEs), drug-related TEAEs, and ≥ grade 3 drug-related hematological TEAEs were 47% vs. 53%, 25% vs. 29%, and 4% vs. 7% in the no prior-chemo and prior-chemo groups, respectively. Incidences of TEAEs (61%), drug-related TEAEs (36%), and ≥ grade 3 drug-related hematological events (12%) were numerically higher in 33 patients who had received two lines of prior chemotherapy. Multivariate analysis showed that two lines of prior chemotherapy, and hemoglobin, platelet, and lactate dehydrogenase values were baseline factors significantly related to ≥ grade 2 platelet count decreased. Safety and effectiveness in patients without concomitant ARATs (*n* = 201) were similar to those in the overall population.

**Conclusion:**

In a real-life setting, radium-223 was well tolerated irrespective of prior chemotherapy, but relatively higher incidences of TEAEs and hematotoxicities were suggested in patients with two lines of prior chemotherapy, possibly reflecting more advanced disease. Radium-223 safety and effectiveness in patients without concomitant ARATs were favorable.

**Supplementary Information:**

The online version contains supplementary material available at 10.1007/s10147-020-01850-3.

## Introduction

As prostate cancer (PC) advances, bone metastasis is common, ultimately occurring in 80–90% of patients [1, 2]. Bone metastasis is associated with reduced quality of life (QoL), and with increased disability, risk of death, and treatment costs [2–11]. Therefore, it is essential to manage bone metastasis effectively as part of metastatic castration-resistant prostate cancer (mCRPC) treatment.

Radium-223 (Ra-223) is the first targeted alpha therapy that prolongs overall survival (OS) in patients with bone mCRPC [12]. In a randomized phase III study in patients with CRPC and symptomatic bone metastases (ALSYMPCA), Ra-223 significantly prolonged OS compared with placebo (median: 14.9 versus 11.3 months; hazard ratio: 0.70, p < 0.001). Ra-223 also significantly prolonged time to first symptomatic skeletal event and improved QoL compared with placebo [12].

Ra-223 was approved in Japan for mCRPC treatment in March 2016 and, to date, has been used in several thousand patients. Because understanding of the safety and usefulness of an approved agent in clinical settings is essential, post-marketing surveillance (PMS) has been conducted in Japan since June 2016, in accordance with Japanese regulations. Primary PMS study results, the safety and effectiveness of Ra-223 in the total study population (*n* = 296), have already been reported, with no new safety concerns identified during the 6-month observation period [13].

The characteristics of patients with CRPC are heterogeneous and, thus, to select the most appropriate treatment, it is necessary to consider the treatment history and characteristics of individual patients. Regarding safety, chemotherapy is generally known to be associated with bone marrow toxicity [14, 15], and hematological treatment-emergent adverse events (TEAEs) are common with Ra-223 treatment [12]. Therefore, the safety and usefulness of Ra-223 based on the status of previous chemotherapy use is of specific interest. Indeed, prior chemotherapy use in non-Japanese patients was shown to lead to an increased risk of hematologic toxicity under Ra-223 treatment in ALSYMPCA [16, 17] and observational [18] studies. This trend is thought to be consistent in Japanese patients, but corroborative data from Japanese patients are lacking. Additionally, we have to consider that patient characteristics often differ due to differences in healthcare systems, reimbursement status, and recommended therapeutic strategies among countries.

It is also essential to assess agents in the context of current real-world treatment strategies. Clinical trials, such as ALSYMPCA and a Japanese phase II study [19], did not include patients who had received prior or concomitant treatment with second-generation androgen-receptor axis-targeted agents (ARATs). Because, nowadays, ARATs are used commonly for PC treatment, it is important to reassess the real-world outcomes of Ra-223 in patients who are treated sequentially with various agents. In particular, data in patients without concomitant use of ARATs are meaningful for evaluating the effects of Ra-223 itself.

In order to obtain further information about the optimal patient characteristics for Ra-223 treatment, and the safety and effectiveness of Ra-223 itself, we performed exploratory subgroup analyses of data from the Japanese PMS study in patients with and without prior chemotherapy, and in patients who did not receive concomitant second-generation ARATs. Also, because the occurrence of hematotoxicity is a safety interest, we investigated patient baseline characteristics contributing to the occurrence of hematotoxicity with Ra-223 treatment.

## Patients and methods

### Study design and patients

This was a non-interventional, prospective, multicenter, single-cohort PMS study performed in Japan (ClinicalTrials.gov NCT02803437). Patients with CRPC and bone metastasis, who were selected for treatment with Ra-223 according to the participating physicians’ routine clinical practice, were eligible for inclusion. The target sample size was 300 patients, and the enrollment period was set at 18 months. The observation period for the primary study was 6 months, from the first administration of Ra-223 to 30 days after the last administration. Ra-223 was administered at a dose of 55 kBq/kg every 4 weeks for up to six cycles.

The institutional review board at each participating center approved the study, which was conducted in accordance with the Declaration of Helsinki and Japanese regulations on Good Post-marketing Study Practice. According to Japanese regulations, written informed consent from patients was not required.

### Endpoints

The primary endpoints were TEAEs and drug-related TEAEs. TEAEs were defined as events which occurred following the first administration of Ra-223 until 30 days after the last administration. An important focus was hematological TEAEs, including anemia, leukopenia, neutropenia, thrombocytopenia, pancytopenia, and other hematological events. The occurrence of skeletal-related events (SREs) was also assessed; SREs included pathological bone fractures, spinal cord compression, events requiring external-beam radiotherapy (EBRT) to relieve skeletal symptoms, events requiring orthopedic surgical intervention, and hypercalcemia. Secondary endpoints included laboratory findings, such as total alkaline phosphatase (t-ALP) and prostate-specific antigen (PSA) levels from baseline.

### Statistical analysis

Statistical analyses were generally exploratory and descriptive in nature. For the analysis based on chemotherapy history, patients were divided into those who had received chemotherapy (docetaxel, cabazitaxel, and other chemotherapy) before the first administration of Ra-223 (defined as prior-chemo group) and those who had not (defined as no prior-chemo group). For the prior-chemo group, results are also shown for the subgroup of patients who had received two lines of prior chemotherapy (defined as two lines of prior-chemo group). In the subgroup analysis of patients who received Ra-223 without concomitant ARATs, only patients with treatment periods for enzalutamide or abiraterone that did not overlap with those for Ra-223 were included (defined as without concomitant ARATs group). Because one patient had received docetaxel overlapping with the Ra-223 treatment period, the patient was excluded from the without concomitant ARATs group. Also, in this manuscript, "(second-generation) ARAT" always refers to abiraterone or enzalutamide. Univariate and multivariate analysis with logistic regression analysis (forward–backward stepwise selection method, significance level of 0.05) for the occurrence of hematotoxicity was performed using baseline patient characteristics and prior treatments as individual risk factors. In this analysis, hematotoxicity was defined as the occurrence of hematological laboratory abnormalities (decline) of ≥ CTCAE grade 2; patients with grade 3/4 abnormalities were too few for meaningful analysis. In addition, to investigate the risk factors for hematotoxicity objectively, hematological laboratory abnormalities, not TEAEs, were used. Corresponding baseline hematological laboratory values were always added into the model of multivariate analysis regardless of the univariate analysis results (e.g. a variable of baseline hemoglobin was always added into the model for investigating the occurrence of decreased hemoglobin), given that these factors were expected to correlate with respective hematotoxicities. Log transformation was performed for baseline variables with heavily-skewed distributions (e.g., t-ALP and PSA). Other statistical analysis details are described in Supplementary Methods.

## Results

### Baseline characteristics and prior and concomitant therapies

Overall, 334 patients were enrolled into the PMS study between June 2016 and November 2017. In November 2019, data for 299 patients were available, and 296 patients were included in the safety and effectiveness analysis sets; three patients who did not receive Ra-223 were excluded. The median observation period was 5.6 (range 1.0–10.6) months.

Baseline characteristics and prior/concomitant therapies are presented in Table 1 according to prior chemotherapy status. Among the 296 patients, 170 (57%) were in the no prior-chemo group, while 126 (43%) were in the prior-chemo group and, of these, 33 (26% of the prior-chemo group, 11% of the overall population) had received two lines of chemotherapy.

**Table 1:** Patient characteristics, previous and concomitant therapy, according to the status of prior chemotherapy

	No prior-chemo Group (*n* = 170)	Prior-chemo Group (*n* = 126)	
		All prior-chemo (*n* = 126)	Two lines of prior-chemo (*n* = 33)
*Patient characteristics*			
Age (years)			
Median (range)	76 (48, 93)	73 (54, 87)	72 (56, 81)
ECOG PS			
0	122 (72%)	85 (68%)	20 (61%)
1	42 (25%)	34 (27%)	11 (33%)
≥ 2	6 (4%)	7 (6%)	2 (6%)
WHO’s cancer pain ladder			
0	127 (75%)	76 (60%)	14 (42%)
1	34 (20%)	33 (26%)	12 (36%)
≥ 2	9 (5%)	17 (13%)	7 (21%)
Gleason Score ^a^			
9–10	79 (47%)	61 (48%)	18 (55%)
Number of bone metastases ^b^			
< 6	58 (34%)	26 (21%)	6 (18%)
6–20	45 (26%)	42 (33%)	9 (27%)
> 20	50 (29%)	46 (37%)	15 (46%)
Superscan	3 (2%)	4 (3%)	1 (3%)

Laboratory value, median (range)						
Hemoglobin (g/dL)	*n* = 162	12.5 (8.5, 16.5)	*n* = 121	11.9 (8.6, 15.4)	*n* = 32	11.1 (8.9, 13.8)
Neutrophil count (× 10^3^/mm^3^)	*n* = 126	3.8 (1.3, 16.1)	*n* = 91	4.5 (1.7, 19.7)	*n* = 26	5.4 (1.7, 19.7)
Platelet count (× 10^3^/mm^3^)	*n* = 160	206 (100, 401)	*n* = 115	221 (78, 514)	*n* = 31	208 (78, 514)
PSA (ng/mL)	*n* = 148	17.3 (0, 1453)	*n* = 104	33.8 (0, 5800)	*n* = 26	131.8 (0, 5015)
Total ALP ^c^ (U/L)	*n* = 155	288 (76, 4761)	*n* = 112	250 (89, 2638)	*n* = 30	282 (146, 1184)
LDH (U/L)	*n* = 153	201 (90, 1851)	*n* = 113	219 (83, 2134)	*n* = 31	236 (106, 1060)

*Previous treatment history for prostate cancer*^d,e^			
Radical therapy	57 (34%)	39 (31%)	11 (33%)
Second-generation ARATs	111 (65%)	98 (78%)	28 (85%)
Enzalutamide	84 (49%)	83 (66%)	26 (79%)
Abiraterone acetate	70 (41%)	66 (52%)	21 (64%)
Docetaxel	0 (0%)	120 (95%)	33 (100%)
Cabazitaxel	0 (0%)	36 (29%)	33 (100%)
EBRT	24 (14%)	33 (26%)	14 (42%)
*Concomitant therapy*^e,f^			
Second-generation ARATs	57 (34%)	37 (29%)	8 (24%)
Enzalutamide	31 (18%)	17 (14%)	3 (9%)
Abiraterone acetate	28 (17%)	24 (19%)	7 (21%)
Bone-modifying agent^g^	69 (41%)	40 (32%)	12 (36%)
EBRT (for palliative or bone)	3 (2%)	6 (5%)	3 (9%)

Data are *n* (%) or median (range)

*ALP* alkaline phosphatase; *ARAT* androgen-receptor axis-targeted agents, *EBRT* external-beam radiation therapy; *ECOG PS* Eastern Cooperative Oncology Group performance status; *LDH* lactate dehydrogenase, *PSA* prostate-specific antigen, *t-ALP* total ALP, *WHO* World Health Organization

^a^At diagnosis

^b^Excluded patients without bone imaging data within 1 year before the start of Ra-223

^c^The presented value is the one calculated by the Japanese Society of Clinical Chemistry (JSCC) method. The normal range of t-ALP measured by JSCC is about 108–321 U/L

^d^Including treatments initiated before the first dose of Ra-223 and also continued after the start of Ra-223

^e^Including overlap

^f^Treatments for which the treatment period overlapped with those of Ra-223

^g^Zoledronic acid, denosumab

The proportion of patients with WHO’s cancer pain ladder score of 0 was numerically smaller (60% vs 75%) and the median PSA value (33.8 vs 17.3 ng/mL) was higher in the prior-chemo group than the no prior-chemo group. The prior-chemo group tended to have more bone metastases; the proportions of patients with extent of disease (EOD) 1 were 21% and 34%, and, for EOD3, were 37% and 29% in the prior-chemo and no prior-chemo groups, respectively. The prior-chemo group was more likely to have previously received ARATs (78% vs 65%) and EBRT (26% vs 14%).

The two lines of prior-chemo group tended to have more advanced disease, indicated by a smaller proportion having PS 0 and WHO’s cancer pain ladder 0, and a higher median PSA value, even compared with the overall prior-chemo group.

Among the overall population (*n* = 296), 201 (68%) patients were treated with Ra-223 without receiving concomitant ARATs. Overall, the baseline characteristics and pattern of prior or concomitant therapies in the without concomitant ARATs group were similar to those of the overall population (Supplementary Table 1).

### Ra-223 exposure

In the overall population, 69% of patients completed six cycles of Ra-223 treatment, and 75% completed five or six cycles. More patients in the no prior-chemo group completed six cycles of Ra-223 (75%) compared to the prior-chemo group (60%). Patients in the two lines of prior-chemo group were notably less likely to complete six cycles of Ra-223 (39%). In the without concomitant ARATs group, 65% of patients completed six cycles of Ra-223.

### Safety

#### TEAEs and drug-related TEAEs

Among the overall population, TEAEs and drug-related TEAEs occurred in 49% and 26% of patients, respectively; the majority of events were grade 1 or 2 in severity.

TEAEs and drug-related TEAEs in the no prior-chemo group and prior-chemo group are summarized in Table 2. The incidences of TEAEs and drug-related TEAEs were 47% vs 53% and 25% vs 29% in the no prior-chemo group and prior-chemo group, and for ≥ grade 3 TEAEs/drug-related TEAEs were 15% vs 23% and 5% vs 9%, respectively. The incidences of these events tended to be even higher in the two lines of prior-chemo group.

**Table 2:** Treatment-emergent adverse events (TEAEs) and drug-related TEAEs

	No prior-chemo Group (*n* = 170)		Prior-chemo Group (*n* = 126)			
			All prior-chemo (*n* = 126)		Two lines of prior-chemo (*n* = 33)	
	TEAE	Drug-related TEAE	TEAE	Drug-related TEAE	TEAE	Drug-related TEAE
	*n * (%)	*n * (%)	*n * (%)	*n * (%)	*n * (%)	*n * (%)
Any event	79 (47%)	42 (25%)	67 (53%)	36 (29%)	20 (61%)	12 (36%)
≥ Grade 3^a^	26 (15%)	9 (5%)	29 (23%)	11 (9%)	12 (36%)	4 (12%)
Serious	24 (14%)	5 (3%)	22 (17%)	7 (6%)	10 (30%)	2 (6%)
Events leading to treatment discontinuation	13 (8%)	5 (3%)	8 (6%)	5 (4%)	4 (12%)	2 (6%)

	Drug-related TEAE					
Drug-related hematological TEAE: PT	Any grade	≥ Grade 3^a^	Any grade	≥ Grade 3^a^	Any grade	≥ Grade 3^a^
Any	29 (17%)	7 (4%)	23 (18%)	9 (7%)	8 (24%)	4 (12%)
Pancytopenia	1 (1%)	1 (1%)	0 –	0 –	0 –	0 –
Anemia (or erythropenia)	20 (12%)	4 (2%)	17 (13%)	7 (6%)	6 (18%)	4 (12%)
Leukopenia	12 (7%)	1 (1%)	9 (7%)	1 (1%)	2 (6%)	0 –
Neutropenia	8 (5%)	2 (1%)	5 (4%)	1 (1%)	1 (3%)	0 –
Thrombocytopenia	9 (5%)	0 –	7 (6%)	3 (2%)	4 (12%)	2 (6%)
Drug-related non-hematological TEAE: PT (Any grade ≥ 1% or Grade 3 > 0%)						
Decreased appetite	3 (2%)	0 –	6 (5%)	0 –	3 (9%)	0 –
Diarrhea	8 (5%)	0 –	6 (5%)	0 –	0 –	0 –
Nausea	3 (2%)	0 –	3 (2%)	1 (1%)	0 –	0 –
Vomiting	2 (1%)	0 –	0 –	0 –	0 –	0 –
Melena	0 –	0 –	1 (1%)	1 (1%)	0 –	0 –
Hematochezia	1 (1%)	0 –	0 –	0 –	0 –	0 –
Nail disorder	0 –	0 –	1 (1%)	0 –	0 –	0 –
Bone pain	3 (2%)	1 (1%)	5 (4%)	2 (2%)	1 (3%)	0 –
Back pain	0 –	0 –	1 (1%)	1 (1%)	0 –	0 –
Malaise	4 (2%)	1 (1%)	2 (2%)	0 –	2 (6%)	0 –
Fatigue	2 (1%)	0 –	0 –	0 –	0 –	0 –
Asthenia	1 (1%)	0 –	0 –	0 –	0 –	0 –
Subdural hematoma	0 –	0 –	1 (1%)	1 (1%)	0 –	0 –
Radiation proctitis	1 (1%)	0 –	0 –	0 –	0 –	0 –

*PT* preferred term by MedDRA ver. 22.0

^a^Worst CTCAE grade (ver. 4.0)

Among drug-related hematological TEAEs, the most common event was anemia (13% of the overall population) (Supplementary Table 2); this trend was similar across groups (Table 2). The incidence of hematological drug-related TEAEs of any grade was similar in the no prior-chemo group and the prior-chemo group (17% vs 18%), and was numerically higher in the two lines of prior-chemo group (24%). Among hematological drug-related TEAEs, the incidences of serious events were 1%, 5%, and 6%, and for events ≥ grade 3 were 4%, 7%, and 12% in the no prior-chemo, prior-chemo, and two lines of prior-chemo groups, respectively.

The incidences of TEAEs and drug-related TEAEs in the without concomitant ARATs group were similar to those for the overall population (Supplementary Table 2).

#### Hematological laboratory abnormalities

Hematological laboratory abnormalities that occurred during Ra-223 treatment are summarized in Table 3. The incidence of ≥ grade 3 hemoglobin decreased was numerically higher in the prior-chemo group (14%) compared with the no prior-chemo group (7%), tending to be even higher in the two lines of prior-chemo group (25%). This trend was also similar for ≥ grade 3 platelet count decreased; the incidences were 1%, 5%, and 13% in the no prior-chemo, prior-chemo, and two lines of prior-chemo groups, respectively. The incidences of ≥ grade 3 neutrophil count decreased were low across all groups (from 0 to 2%).

**Table 3:** Hematological laboratory abnormalities that occurred during Ra-223 treatment, by worst CTCAE grade^a^

Laboratory parameters	CTCAE grade^a^	No prior-chemo group	Prior-chemo group		Without concomitant ARATs group	Overall population
			All prior-chemo	2 lines of prior-chemo		
		*n* (%)	*n* (%)	*n* (%)	*n* (%)	*n* (%)
Anemia (hemoglobin decreased)	N^b^	162	121	32	195	283
	Grade 2	31 (19%)	22 (18%)	8 (25%)	39 (20%)	53 (19%)
	Grade 3	12 (7%)	17 (14%)	8 (25%)	23 (12%)	29 (10%)
	Grade 4	0 –	0 –	0 –	0 –	0 –
Neutrophil count decreased	N^b^	126	91	26	145	217
	Grade 2	13 (10%)	7 (8%)	3 (12%)	14 (10%)	20 (9%)
	Grade 3	1 (1%)	0 –	0 –	0 –	1 (< 1%)
	Grade 4	2 (2%)	1 (1%)	0 –	1 (1%)	3 (1%)
Platelet count decreased	N^b^	160	115	31	189	275
	Grade 2	5 (3%)	4 (3%)	1 (3%)	7 (4%)	9 (3%)
	Grade 3	2 (1%)	2 (2%)	1 (3%)	2 (1%)	4 (1%)
	Grade 4	0 –	4 (3%)	3 (10%)	3 (2%)	4 (1%)

^a^Tabulated with CTCAE grades for the lowest laboratory values from after the start of the drug to 30 days after the last dose

^b^No. of analyzed patients

The overall incidence of hematological laboratory abnormalities in the without concomitant ARATs group was similar to that in the overall population.

#### Related baseline characteristics for ≥ grade 2 hematological laboratory abnormalities

Patient baseline characteristics related to the occurrence of ≥ grade 2 hematological abnormalities are shown in Table 4. Multivariate analysis showed that, low hemoglobin high PSA, and high t-ALP at baseline were significantly related to the occurrence of ≥ grade 2 hemoglobin decrease. With respect to the occurrence of ≥ grade 2 platelet count decrease, low hemoglobin, low platelet count, high LDH, and two lines of prior chemotherapy were significantly related. Regarding the occurrence of decreased neutrophil count, because the availability of the model was low (0.635) due to missing data, this result was considered as a reference only.

**Table 4:** Univariate and multivariate analyses for the incidence of ≥ grade 2 hematological laboratory abnormalities (logistic regression analysis)

Variable	Univariate analysis			Multivariate analysis^g^		
	Odds ratio	Odds ratio (95% CI)	p-value	Odds ratio	Odds ratio (95% CI)	p-value
(A) Decrease in hemoglobin of ≥ CTCAE grade 2						
Age (years)^a^	1.04	(1.01, 1.08)	0.014			
Baseline hemoglobin (g/dL)^a^	0.29	(0.22, 0.40)	< 0.001	0.32	(0.22, 0.47)	< 0.001
Baseline log_10_ (PSA, ng/mL)^a^	2.66	(1.83, 3.86)	< 0.001	1.93	(1.20, 3.09)	0.006
Baseline log_10_ (t-ALP, U/L)^a^	9.44	(3.81, 23.41)	< 0.001	4.40	(1.20, 16.09)	0.025
Baseline log_10_ (LDH, U/L)^a^	34.84	(6.12, 198.32)	< 0.001			
Number of bone metastases: ≤ 20 (vs. > 20)^b^	0.37	(0.21, 0.63)	< 0.001			
ECOG PS: ≥ 1 (vs. 0)	2.01	(1.19, 3.38)	0.009			
WHO’s Cancer Pain ladder: ≥ 1 (vs. 0)^c^	1.94	(1.16, 3.25)	0.011			
Prior treatment with chemotherapy: yes (vs. no)^d^	1.46	(0.89, 2.39)	0.131			
Prior treatment with 2 lines of chemotherapy: yes (vs. no)^e^	3.14	(1.54, 6.39)	0.002			
Treatment line of Ra-223: 2nd line (vs. first line)^f^	0.99	(0.50, 1.92)	0.965			
Treatment line of Ra-223: 3rd line or later (vs. first line)^f^	2.09	(1.14, 3.81)	0.017			
(B) Decrease in platelet count of ≥ CTCAE grade 2						
Baseline hemoglobin (g/dL)^a^	0.57	(0.42, 0.79)	< 0.001	0.63	(0.44, 0.92)	0.016
Baseline platelet count (× 10^4^/mm^3^)^a^	0.89	(0.81, 0.96)	0.005	0.90	(0.82, 0.98)	0.022
Baseline log_10_ (t-ALP, U/L)^a^	8.14	(2.44, 27.18)	< 0.001			
Baseline log_10_ (LDH, U/L)^a^	226.54	(24.22, 2118.75)	< 0.001	65.89	(6.21, 699.25)	< 0.001
Prior treatment with 2 line of chemotherapy: yes (vs. no)^e^	4.36	(1.71, 11.09)	0.002	3.33	(1.05, 10.54)	0.041
(C) Decrease in neutrophil count of ≥ CTCAE grade 2						
Baseline neutrophil count (× 10^3^/mm^3^)^a^	0.64	(0.49, 0.85)	0.002	0.62	(0.46, 0.84)	0.002
Baseline log_10_ (PSA, ng/mL)^a^	1.62	(1.05, 2.50)	0.031			
Baseline log_10_ (t-ALP, U/L)^a^	3.43	(1.12, 10.53)	0.031			
Prior treatment with chemotherapy: yes (vs. no)^d^	0.84	(0.39, 1.79)	0.646			

Variables with 15% or more missing, and variables with an extreme distribution (95% or more of the data biased to one level) were excluded from the study. If there were strong correlations between two variables, only one variable from those variables was included into the logistic regression models

(A) Availability: 0.750; Adjusted analysis using variables in univariate analysis resulted in c-statistic of 0.904

(B) Availability: 0.848; Adjusted analysis using variables in univariate analysis resulted in c-statistic of 0.884

(C) Availability: 0.635; Adjusted analysis using variables in univariate analysis resulted in c-statistic of 0.768

^a^Per unit

^b^Superscan is excluded because its clinical condition is different from others

^c^The results of multivariate analyses and c-statistic (0.904) in adjusted analyses were the same when the variable was exchanged from “WHO’s Cancer Pain ladder: ≥1 (vs. 0)” to “WHO’s Cancer Pain ladder: ≥2 (vs. ≤1)” in the models. Consequently, only one model (including WHO’s Cancer Pain ladder ≥1 [vs. 0]) is described in the table

^d^Docetaxel and/or cabazitaxel and/or other chemotherapy

^e^Docetaxel and cabazitaxel

^f^Defined as the order of which Ra-223 was used among CRPC treatment with drugs including Ra-223, enzalutamide, abiraterone acetate, docetaxel and cabazitaxel. Ra-223 is 1st line: Ra-223 was used first among the above five agents (no agent is used before Ra-223 treatment); Ra-223 is 2nd line: One of the other four agents above was used first, after which Ra-223 was used; Ra-223 is 3rd line or later: Two or more of the other four agents above were used, after which Ra-223 was used

^g^Logistic regression analysis (forward–backward selection stepwise method), significance level of 5%

#### Skeletal-related events and fractures

A drug-related SRE occurred in one patient in the overall population (0.3%); the patient was in the prior-chemo group (0.8%) and also belonged to the without concomitant ARATs group (0.5%). The SRE was an event requiring EBRT.

During the observation period, a total of five fractures were reported, all of which were judged unrelated to Ra-223 by investigators; fractures included femoral (*n* = 2), rib (*n* = 1), femoral neck (*n* = 1), and pathological bone (*n* = 1). Only one of the five patients had received concomitant bone-modifying agents.

### Effectiveness

#### Percent changes in t-ALP and PSA over 24 weeks

**Fig. 1:**
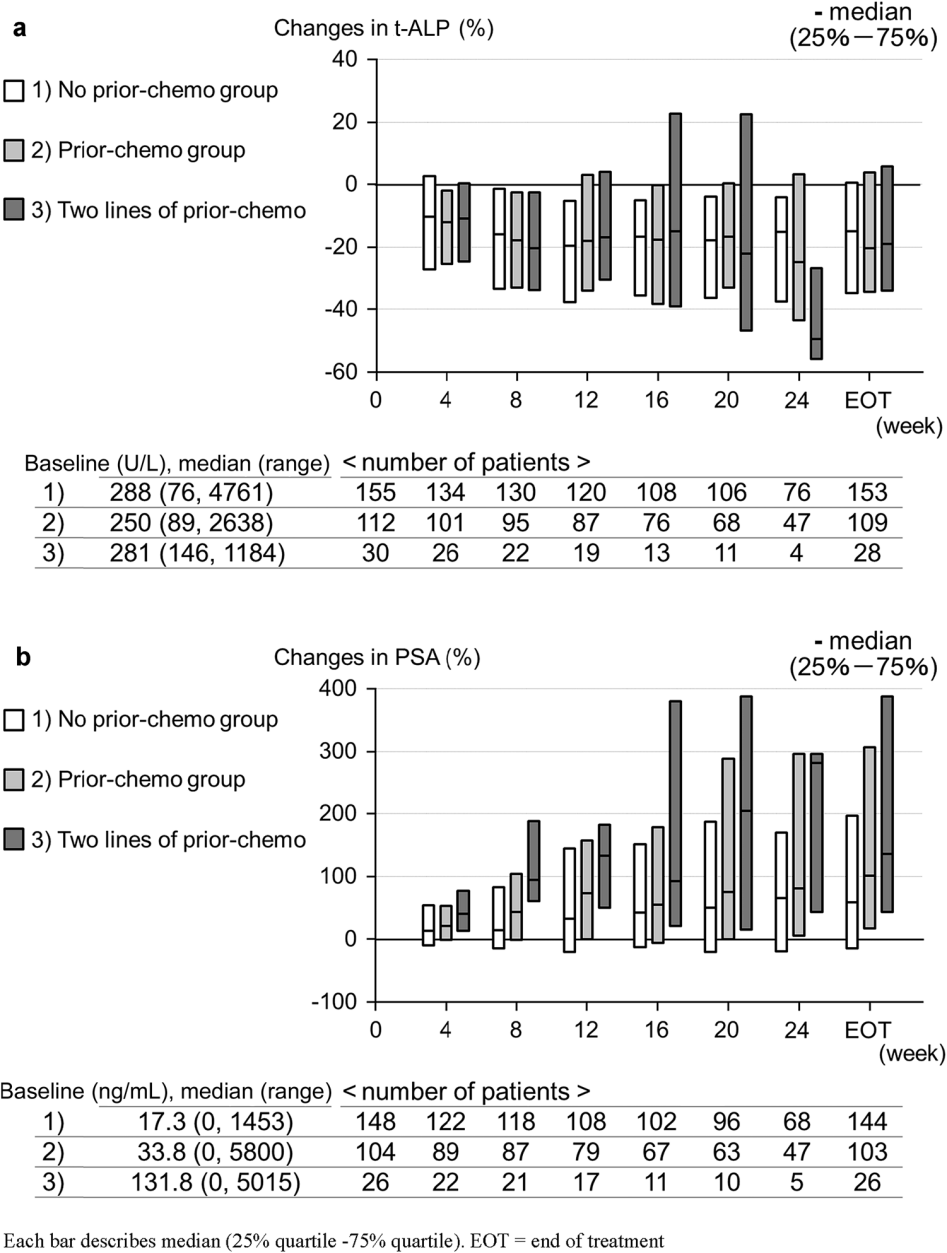
Percent changes in (a) t-ALP and (b) PSA from baseline over 24 weeks by prior chemotherapy status

Percent changes from baseline in t-ALP and PSA over 24 weeks are summarized in Fig. 1, Supplementary Fig. 1, and Supplementary Table 3.

t-ALP decreased in most patients across all groups during the observation period and the transition of changes was not evidently different across groups. t-ALP reductions were observed from week 4 after the first administration of Ra-223 and were sustained throughout the observation period.

Median percent changes in PSA from baseline were shown to increase in all groups, although the increases were relatively smaller in the no prior-chemo group compared to those in the prior-chemo group; the increases tended to be larger in patients who had two lines of prior chemotherapy. The transition of changes in PSA over 24 weeks in the without concomitant ARATs group was similar to that for the overall population.

#### t-ALP and PSA changes at Week 12 by category

When categorized by extent of change at week 12, any decline in t-ALP was observed in 63–82% of patients across groups (Supplementary Table 3).

At week 12, any decline in PSA was observed in 34%, 23%, and 12% of patients in the no prior-chemo, prior-chemo, and two lines of prior-chemo groups, respectively. The proportions of patients with any decline in PSA at week 12 were similar in the without concomitant ARATs group and overall population.

## Discussion

This PMS study is the largest prospective observational study of Ra-223 conducted in Japan. The primary results for the overall population (*n* = 296) have been published previously [13]. Considering that Ra-223 has been used for patients with various backgrounds in Japanese clinical settings, we performed exploratory analyses to investigate its safety and effectiveness in subgroups by status of prior chemotherapy or concurrent use of ARATs.

In this study, 43% of patients had received chemotherapy before Ra-223 treatment. In the prior-chemo group, some baseline patient characteristics suggesting more advanced PC were observed compared to the no prior-chemo group, including high PSA values and more bone metastases. Additionally, other treatments, including ARATs and EBRT, were used more frequently before Ra-223 in the prior-chemo group, indicating that this population was heavily treated. Overall, Ra-223 was well tolerated in all groups. However, the incidences of TEAEs/drug-related TEAEs with serious or ≥ grade 3 events, and ≥ grade 3 hematological TEAEs, were numerically higher in the prior-chemo group than the no prior-chemo group. Notably, baseline characteristics aligned with advanced PC and a more frequent incidence of TEAEs were evident in the two lines of prior-chemo group, suggesting that more adverse events were presumably due to more advanced disease stage in these patients.

In multivariate analysis, the selected significant factors for hematological abnormalities were low hematological laboratory values at baseline or baseline characteristics suggesting advanced primary disease. While some selected factors were different from those in ALSYMPCA [17], the trend was not contradictory. It is important to understand that differences in era, patient population, and study design may affect the results; for example, cabazitaxel had not yet been approved at initiation of ALSYMPCA. A history of docetaxel use was a significant factor for thrombocytopenia in ALSYMPCA [17], and prior treatment with two lines of chemotherapy was a selected factor for decreased platelet count in the current study. In line with the fact that the incidence of overall TEAEs was especially higher in the two lines of prior-chemo group compared to other groups, this highlights the importance of monitoring patients and paying attention to the occurrence of adverse events particularly in Japanese patients with a history of intensive chemotherapy.

While the decline in t-ALP from baseline was observed in many patients across all groups, the median of percent change in PSA during the Ra-223 treatment period was relatively higher in the prior-chemo group. Notably, the proportion of patients who completed six cycles of Ra-223 was smaller in the prior-chemo group, especially in the two lines of prior-chemo group. Given that some patients might discontinue Ra-223 when PSA increases are unbearable, PSA dynamics might have influenced, partially at least, the Ra-223 completion rate. Unfortunately, because detailed information about disease progression, as a reason for discontinuation, was not obtained in this study, the profile of disease progression was unclear. However, as recommended by guidelines [20], the assessment of treatment effect and decisions to change treatment should be performed comprehensively.

Consensus about the best CRPC treatment sequence has not yet been reached [21, 22], and the best sequence with Ra-223 and other agents also remains unclear. From the sub-analysis of ALSYMPCA, it was shown that, compared with placebo, the Ra-223 survival benefit was sustained regardless of prior chemotherapy [16], and that Ra-223 treatment did not affect subsequent chemotherapy [23]. Also, considering these reports, current subgroup results indicating tendencies of fewer TEAEs and a higher Ra-223 completion rate in the no prior-chemo group might partially suggest that Ra-223 treatment before chemotherapy may be beneficial to patients; however, to reach decisive conclusions, findings regarding the effect of each treatment sequence on overall survival are awaited.

We also investigated the safety and effectiveness of Ra-223 in patients without concomitant use of second-generation ARATs. The fact that most outcomes in this subgroup were similar to those in the overall population provides encouragement that the safety and effectiveness of Ra-223 itself in current real-world settings is sustained without concomitant life-prolonging CRPC treatment, and in patients receiving various sequential CRPC treatments.

There are some limitations to the results, including small sample size and single-cohort study design. Subgroup analysis results from a single cohort should be interpreted cautiously, because they are often attributed to differences in baseline characteristics. However, our analysis reflects real-world patients and should help to develop treatment strategies in clinical practice without influence from patient selection or interventions. Also, although the effect of treatment on QoL is an important aspect for patients, endpoints related to QoL were not included in the protocol of this study because PMS is a survey to mainly investigate the drug’s safety. Prospective investigations about QoL, as well as quality adjusted life-year, in Japanese patients treated with Ra-223 would be expected in the future. Follow-up for this study, up to 3 years, is ongoing and future findings, including prognosis and fracture information, will further strengthen the findings from this exploratory analysis.

## Supplementary Information

Below is the link to the electronic supplementary material.

Supplementary file 1 (DOCX 196 KB)
