# Supplementary material for: Real-world safety and effectiveness of radium-223 in Japanese patients with castration-resistant prostate cancer (CRPC) and bone metastasis: exploratory analysis, based on the results of post-marketing surveillance, according to prior chemotherapy status and in patients without concomitant use of second-generation androgen-receptor axis-targeted agents
Source: Int J Clin Oncol. 2021 Feb 11;26(4):753–63. doi: 10.1007/s10147-020-01850-3 (PMC7979648; doi:10.1007/s10147-020-01850-3)
Supplement: Supplementary file 1 — (DOCX 196 KB) [file 10147_2020_1850_MOESM1_ESM.docx]

**Supplementary Material**

**Supplementary Methods (Statistical Analysis)**

**Supplementary Table 1. Patient characteristics, previous and concomitant therapy for the without concomitant ARATs group and overall population**

**Supplementary Table 2. Treatment-emergent adverse events (TEAEs) and drug-related TEAEs in the** **without concomitant ARATs group and overall population**

**Supplementary Table 3.** **Changes in total alkaline phosphatase (t-ALP) and prostate-specific antigen (PSA) under Ra-223 treatment**

**Supplementary Fig. 1. Percent changes in (a) t-ALP and (b) PSA from baseline over 24 weeks in the without concomitant ARATs group and overall population**

**Supplementary Methods: Statistical Analysis**

All enrolled patients who received at least one cycle of Ra-223 were included in the safety analysis set. Enrolled patients who met the inclusion criteria were included in the effectiveness analysis set. TEAEs were coded according to MedDRA Version 22.0. The severity of TEAEs was summarized according to CTCAE Version 4.0 - Japanese Clinical Oncology Group. The definition of SAE is as follows: results in death, is life-threatening, requires inpatient hospitalization or prolongation of existing hospitalization, results in persistent or significant disability or incapacity, is a congenital anomaly or birth defect, or is medically important.

All statistical analysis was performed with SAS version 9.4 (SAS Institute Inc. Cary, NC).

**Supplementary Table 1. Patient characteristics, previous and concomitant therapy in the without concomitant ARATs group and overall population**

|  | | Without concomitant ARATs group (n = 201) | | |  | | Overall population | | | | | |
| --- | --- | --- | --- | --- | --- | --- | --- | --- | --- | --- | --- | --- |
|  |  |  |  |  |  | | | (n = 296) | | | | |
| Age (years) |  |  |  | |  | |  | | | |  | |
| median (range) |  |  | 75 (48, 93) | |  | |  | | | | 75 (48, 93) | |
| ECOG PS |  |  |  |  |  | | |  | | |  |  |
| 0 |  |  | 141 | (70%) |  | | |  | | | 207 | (70%) |
| 1 |  |  | 51 | (25%) |  | | |  | | | 76 | (26%) |
| ≥ 2 |  |  | 9 | (4%) |  | | |  | | | 13 | (4%) |
| WHO’s cancer pain ladder |  |  |  |  |  | | |  | | |  |  |
| 0 |  |  | 133 | (66%) |  | | |  | | | 203 | (69%) |
| 1 |  |  | 47 | (23%) |  | | |  | | | 67 | (23%) |
| ≥ 2 |  |  | 21 | (10%) |  | | |  | | | 26 | (9%) |
| Gleason Score ^a^ |  |  |  |  |  | | |  | | |  |  |
| 9-10 |  |  | 94 | (47%) |  | | |  | | | 140 | (47%) |
| Number of bone metastases ^b^ |  |  |  |  |  | | |  | | |  |  |
| <6 |  |  | 51 | (25%) |  | | |  | | | 84 | (28%) |
| 6-20 |  |  | 63 | (31%) |  | | |  | | | 87 | (29%) |
| >20 |  |  | 66 | (33%) |  | | |  | | | 96 | (32%) |
| Superscan |  |  | 7 | (3%) |  | | |  | | | 7 | (2%) |
| Hemoglobin (g/dL) | | n = 195 | 11.9 (8.5, 16.5) | |  | | n = 283 | | | | 12.1 (8.5, 16.5) | |
| Neutrophil count (× 10^3^/mm^3^) | | n = 145 | 4.1 (1.7, 19.7) | |  | | n = 217 | | | | 4.0 (1.3, 19.7) | |
| Platelet count (× 10^3^/mm^3^) | | n = 189 | 207 (78, 514) | |  | | n = 275 | | | | 212 (78, 514) | |
| PSA (ng/mL) | | n = 171 | 20.7 (0, 5800) | |  | | n = 252 | | | | 20.7 (0, 5800) | |
| Total ALP ^c^ (U/L) | | n = 185 | 288 (76, 4761) | |  | | n = 267 | | | | 271 (76, 4761) | |
| LDH (U/L) | | n = 184 | 204 (90, 2134) | |  | | n = 266 | | | | 206 (83, 2134) | |
| **Previous treatment history for prostate cancer** ^d,e^ | | | | | | | | | | | | |
| Radical therapy | |  | 70 | (35%) |  | | |  | | | 96 | (32%) |
| Second-generation ARATs | |  | 121 | (60%) |  | | |  | | | 209 | (71%) |
| Enzalutamide | |  | 99 | (49%) |  | | |  | | | 167 | (56%) |
| Abiraterone acetate | |  | 79 | (39%) |  | | |  | | | 136 | (46%) |
| Second generation ARATs completed before the first dosing of Ra-223 ^f^ | | | 121 | (60%) |  | | |  | | | 160 | (54%) |
| 1 ARAT | |  | 64 | (32%) |  | | |  | | | 102 | (34%) |
| 2 ARATs | |  | 57 | (28%) |  | | |  | | | 58 | (20%) |
| Docetaxel |  |  | 83 | (41%) |  | | |  | | | 120 | (41%) |
| Cabazitaxel |  |  | 27 | (13%) |  | | |  | | | 36 | (12%) |
| EBRT (for palliative or bone) |  |  | 39 | (19%) |  | | |  | | | 57 | (19%) |
| **Combination of previous treatment histories** ^d^ | | | | | | | | | | | | |
| Chemotherapy: no, ARATs: no | | | 54 | (27%) | |  | | |  | | 60 | (20%) |
| Chemotherapy: no, ARATs: yes | | | 61 | (30%) | |  | | |  | | 113 | (38%) |
| Chemotherapy: yes, ARATs: no | | | 26 | (13%) | |  | | |  | | 27 | (9%) |
| Chemotherapy: yes, ARATs: yes | | | 60 | (30%) | |  | | |  | | 96 | (32%) |
| **Concomitant therapy** ^e,g^ | | | | | | | | | | | | |
| Second-generation ARATs | | | 0 | – | |  | | | |  | 94 | (32%) |
| Enzalutamide |  | | 0 | – | |  | | | |  | 48 | (16%) |
| Abiraterone acetate |  | | 0 | – | |  | | | |  | 52 | (18%) |
| Bone-modifying agent ^h^ |  | | 72 | (36%) | |  | | | |  | 109 | (37%) |
| EBRT (for palliative or bone) | | | 5 | (2%) | |  | | | |  | 9 | (3%) |

Data are n (%) or median (range)

^a^ At diagnosis

^b^ Excluded patients without bone imaging data within 1 year before the start of Ra-223

^c^ The presented value is the one calculated by the Japanese Society of Clinical Chemistry (JSCC) method. The normal range of t-ALP measured by JSCC is about 108–321 U/L

^d^ Including treatments initiated before the first dose of Ra-223 and also continued after the start of Ra-223

^e^ Including overlap

^f^ Excluded treatments initiated before the first dose of Ra-223 and also continued after the start of Ra-223

^g^ Treatments for which the treatment period overlapped with those of Ra-223

^h^ Zoledronic acid, denosumab

ALP = alkaline phosphatase; ARAT = androgen-receptor axis-targeted agent; EBRT = external-beam radiation therapy; ECOG PS = Eastern Cooperative Oncology Group performance status; LDH = lactate dehydrogenase; PSA = prostate-specific antigen; t-ALP = total ALP; WHO = World Health Organization

**Supplementary Table 2. Treatment-emergent adverse events (TEAEs) and drug-related TEAEs in the without concomitant ARATs group and overall population**

|  | Without concomitant ARATs group (n = 201) | | | | Overall population (n = 296) | | | | |
| --- | --- | --- | --- | --- | --- | --- | --- | --- | --- |
|  | TEAE | | drug-related TEAE | | TEAE | | drug-related TEAE | |  |
|  | n | (%) | n | (%) | n | (%) | n | (%) |  |
| Any event | 102 | (51%) | 54 | (27%) | 146 | (49%) | 78 | (26%) |  |
| ≥ Grade 3^a^ | 38 | (19%) | 16 | (8%) | 55 | (19%) | 20 | (7%) |  |
| Serious | 33 | (16%) | 11 | (5%) | 46 | (16%) | 12 | (4%) |  |
| Events leading to treatment discontinuation | 15 | (7%) | 6 | (3%) | 21 | (7%) | 10 | (3%) |  |
|  | Drug-related TEAE | | | | | | | |  |
| Drug-related hematological TEAE: PT | Any grade | | ≥ Grade 3^a^ | | Any grade | | ≥ Grade 3^a^ | |  |
| Any | 37 | (18%) | 13 | (7%) | 52 | (18%) | 16 | (5%) |  |
| Pancytopenia | 1 | (1%) | 1 | (1%) | 1 | (<1%) | 1 | (<1%) |  |
| Anemia (or erythropenia) | 29 | (14%) | 9 | (5%) | 37 | (13%) | 11 | (4%) |  |
| Leukopenia | 14 | (7%) | 1 | (1%) | 21 | (7%) | 2 | (1%) |  |
| Neutropenia | 10 | (5%) | 2 | (1%) | 13 | (4%) | 3 | (1%) |  |
| Thrombocytopenia | 15 | (7%) | 3 | (1%) | 16 | (5%) | 3 | (1%) |  |
| Drug-related non-hematological TEAE:  PT (Any grade >1% or Grade 3 >0%) | Any grade | | ≥ Grade 3^a^ | | Any grade | | ≥ Grade 3^a^ | |  |
| Decreased appetite | 3 | (1%) | 0 | – | 9 | (3%) | 0 | – |  |
| Diarrhea | 10 | (5%) | 0 | – | 14 | (5%) | 0 | – |  |
| Nausea | 5 | (2%) | 1 | (1%) | 6 | (2%) | 1 | (<1%) |  |
| Vomiting | 2 | (1%) | 0 | – | 2 | (1%) | 0 | – |  |
| Melena | 1 | (1%) | 1 | (1%) | 1 | (<1%) | 1 | (<1%) |  |
| Hematochezia | 1 | (1%) | 0 | – | 1 | (<1%) | 0 | – |  |
| Nail disorder | 0 | – | 0 | – | 1 | (<1%) | 0 | – |  |
| Bone pain | 6 | (3%) | 3 | (1%) | 8 | (3%) | 3 | (1%) |  |
| Back pain | 1 | (1%) | 1 | (1%) | 1 | (<1%) | 1 | (<1%) |  |
| Malaise | 3 | (1%) | 0 | – | 6 | (2%) | 1 | (<1%) |  |
| Fatigue | 1 | (1%) | 0 | – | 2 | (1%) | 0 | – |  |
| Asthenia | 0 | – | 0 | – | 1 | (<1%) | 0 | – |  |
| Subdural hematoma | 1 | (1%) | 1 | (1%) | 1 | (<1%) | 1 | (<1%) |  |
| Radiation proctitis | 1 | (1%) | 0 | – | 1 | (<1%) | 0 | – |  |

^a^ Worst CTCAE grade (ver. 4.0)

ARAT = androgen-receptor axis-targeted agent; PT = preferred term by MedDRA ver. 22.0

**Supplementary Table 3. Changes in total alkaline phosphatase (t-ALP) and prostate-specific antigen (PSA) under Ra-223 treatment**

**a) t-ALP**

|  | | No prior-chemo group  (n = 170) | | | Prior-chemo group (n = 126) | | | | | | Without concomitant ARATs group (n = 201) | | | Overall population  (n = 296) | | |
| --- | --- | --- | --- | --- | --- | --- | --- | --- | --- | --- | --- | --- | --- | --- | --- | --- |
|  |  |  |  |  | All prior-chemo (n = 126) | | | 2 lines of prior-chemo (n = 33) | | |  |  |  |  |  |  |
|  |  | n | median | range | n | median | (range) | n | median | (range) | n | median | (range) | n | median | (range) |
| Baseline (U/L) | | 155 | 288 | (76,  4761) | 112 | 250 | (89,  2638) | 30 | 282 | (146,  1184) | 185 | 288 | (76,  4761) | 267 | 271 | (76,  4761) |
| Percent change  from baseline  (%) | week 4 | 134 | -10.3 | (-72.4,  115.7) | 101 | -12.1 | (-55.2,  131.9) | 26 | -10.9 | (-42.4,  55.2) | 161 | -12.0 | (-72.4,  131.9) | 235 | -11.1 | (-72.4, 131.9) |
|  | week 8 | 130 | -15.9 | (-78.0,  263.2) | 95 | -17.9 | (-73.8,  236.1) | 22 | -20.3 | (-61.7,  101.4) | 153 | -18.3 | (-78.0,  263.2) | 225 | -16.7 | (-78.0,  263.2) |
|  | week 12 | 120 | -19.5 | (-82.4,  459.4) | 87 | -18.0 | (-76.5,  203.9) | 19 | -16.8 | (-63.2,  196.7) | 139 | -20.4 | (-82.4,  459.4) | 207 | -19.4 | (-82.4,  459.4) |
|  | week 16 | 108 | -16.6 | (-83.2,  73.8) | 76 | -17.6 | (-79.2,  247.3) | 13 | -15.0 | (-64.4,  247.3) | 123 | -18.7 | (-83.2,  113.5) | 184 | -17.1 | (-83.2,  247.3) |
|  | week 20 | 106 | -17.7 | (-85.2,  382.1) | 68 | -16.7 | (-80.6,  188.3) | 11 | -22.2 | (-72.8,  188.3) | 112 | -18.4 | (-85.2, 382.1) | 174 | -17.7 | (-85.2,  382.1) |
|  | week 24 | 76 | -15.1 | (-84.7,  146.6) | 47 | -24.7 | (-85.7,  125.6) | 4 | -49.5 | (-57.1,  -8.7) | 76 | -22.6 | (-85.7,  146.6) | 123 | -20.0 | (-85.7,  146.6) |
|  | end of treatment | 153 | -15.0 | (-84.7,  459.4) | 109 | -20.4 | (-85.7,  268.1) | 28 | -19.0 | (-72.8,  182.9) | 181 | -17.6 | (-85.7, 459.4) | 262 | -15.8 | (-85.7,  459.4) |
| Percent change  at Week 12, n (%) | No. of analyzed patients ^a^ | n=120 | | | n=87 | | | n=19 | | | n=139 | | | n=207 | | |
|  | < 0% | 82% | | | 72% | | | 63% | | | 76% | | | 78% | | |
|  | [≤ -30%] | [33%] | | | [28%] | | | [26%] | | | [34%] | | | [30%] | | |
| Percent changes in t-ALP and PSA at Week 12, n (%) | No. of analyzed patients ^b^ | n=107 | | | n=76 | | | n=16 | | | n=120 | | | n=183 | | |
|  | ALP<0% and PSA<0% | 33% | | | 17% | | | 13% | | | 26% | | | 26% | | |
|  | ALP<0% and PSA>0% | 50% | | | 57% | | | 56% | | | 53% | | | 53% | | |
|  | ALP>0% and PSA<0% | 1% | | | 5% | | | 0% | | | 3% | | | 3% | | |
|  | ALP>0% and PSA>0% | 16% | | | 21% | | | 31% | | | 19% | | | 18% | | |

^a^ Patients with data for ALP at week 12 were included

^b^ Patients with both data for ALP and PSA at week 12 were included

**b) PSA**

|  | | No prior-chemo group  (n = 170) | | | | | | Prior-chemo group (n = 126) | | | | | | | | | | | | | | Without concomitant ARATs group (n = 201) | | | | | | | Overall population  (n = 296) | | | | | |
| --- | --- | --- | --- | --- | --- | --- | --- | --- | --- | --- | --- | --- | --- | --- | --- | --- | --- | --- | --- | --- | --- | --- | --- | --- | --- | --- | --- | --- | --- | --- | --- | --- | --- | --- |
|  |  |  |  |  |  |  |  | All prior-chemo (n = 126) | | | | | | | 2 lines of prior-chemo (n = 33) | | | | | | |  |  |  |  |  |  |  |  |  |  |  |  |  |
|  |  | n | | median | | (range) | | n | | median | | (range) | | n | | | median | | (range) | | n | | | median | | (range) | | n | | | median | | (range) | |
| Baseline (ng/mL) | | 148 | | 17.3 | | (0.0,  1452.6) | | 104 | | 33.8 | | (0.0,  5800.4) | | 26 | | | 131.8 | | (0.0,  5015.3) | | 171 | | | 20.7 | | (0.0,  5800.4) | | 252 | | | 20.7 | | (0.0,  5800.4) | |
| Percent change  from baseline  (%) | week 4 | | 122 | | 13.8 | | (-98.4, 322.5) | | 89 | | 20.8 | | (-96.9, 921.4) | | 22 | | | 40.5 | | (-45.0, 178.5) | | 142 | | | 20.4 | | (-86.7, 921.4) | | 211 | | | 17.2 | | (-98.4,  921.4) |
|  | week 8 | | 118 | | 14.8 | | (-99.1, 685.8) | | 87 | | 43.8 | | (-98.5, 3760.9) | | 21 | | | 94.5 | | (-39.6, 3760.9) | | 139 | | | 31.3 | | (-93.3, 3760.9) | | 205 | | | 27.4 | | (-99.1, 3760.9) |
|  | week 12 | | 108 | | 32.2 | | (-99.4, 2101.1) | | 79 | | 73.2 | | (-99.2, 2291.3) | | 17 | | | 132.8 | | (-56.6, 1011.8) | | 123 | | | 46.8 | | (-98.7, 2291.3) | | 187 | | | 46.0 | | (-99.4, 2291.3) |
|  | week 16 | | 102 | | 42.2 | | (-99.5,  4203.2) | | 67 | | 54.5 | | (-98.5, 3328.6) | | 11 | | | 92.4 | | (-54.5, 1850.0) | | 110 | | | 57.0 | | (-97.4, 4203.2) | | 169 | | | 49.3 | | (-99.5, 4203.2) |
|  | week 20 | | 96 | | 49.8 | | (-99.7, 3202.2) | | 63 | | 75.5 | | (-98.1, 3335.7) | | 10 | | | 204.5 | | (-54.5, 831.5) | | 100 | | | 78.2 | | (-99.7, 3335.7) | | 159 | | | 56.8 | | (-99.7, 3335.7) |
|  | week 24 | | 68 | | 65.9 | | (-99.7, 2759.0) | | 47 | | 80.8 | | (-98.7, 4821.4) | | 5 | | | 282.2 | | (-58.5, 331.6) | | 73 | | | 72.6 | | (-99.7, 4821.4) | | 115 | | | 72.6 | | (-99.7, 4821.4) |
|  | end of treatment | | 144 | | 58.6 | | (-99.7, 4203.2) | | 103 | | 101.7 | | (-98.7, 4821.4) | | 26 | | | 135.9 | | (-58.5, 3760.9) | | 167 | | | 95.7 | | (-99.7, 4821.4) | | 247 | | | 83.0 | | (-99.7, 4821.4) |
| Percent change  at Week 12, n (%) | No. of analyzed patients ^c^ | | n=108 | | | | | | n=79 | | | | | | | n=17 | | | | | | | n=123 | | | | | | | n=187 | | | | |
|  | < 0% | | 34% | | | | | | 23% | | | | | | | 12% | | | | | | | 29% | | | | | | | 29% | | | | |
|  | [≤ -30%] | | [19%] | | | | | | [16%] | | | | | | | [12%] | | | | | | | [17%] | | | | | | | [18%] | | | | |
|  | 0% ≤ < 100% | | 36% | | | | | | 39% | | | | | | | 29% | | | | | | | 33% | | | | | | | 37% | | | | |
|  | 100% ≤ | | 30% | | | | | | 38% | | | | | | | 59% | | | | | | | 38% | | | | | | | 33% | | | | |

^c^ Patients with data for PSA at week 12 were included

**Supplementary Fig. 1. Percent changes in (a) t-ALP and (b) PSA from baseline over 24 weeks in the without concomitant ARATs group and overall population**

**a)**

**
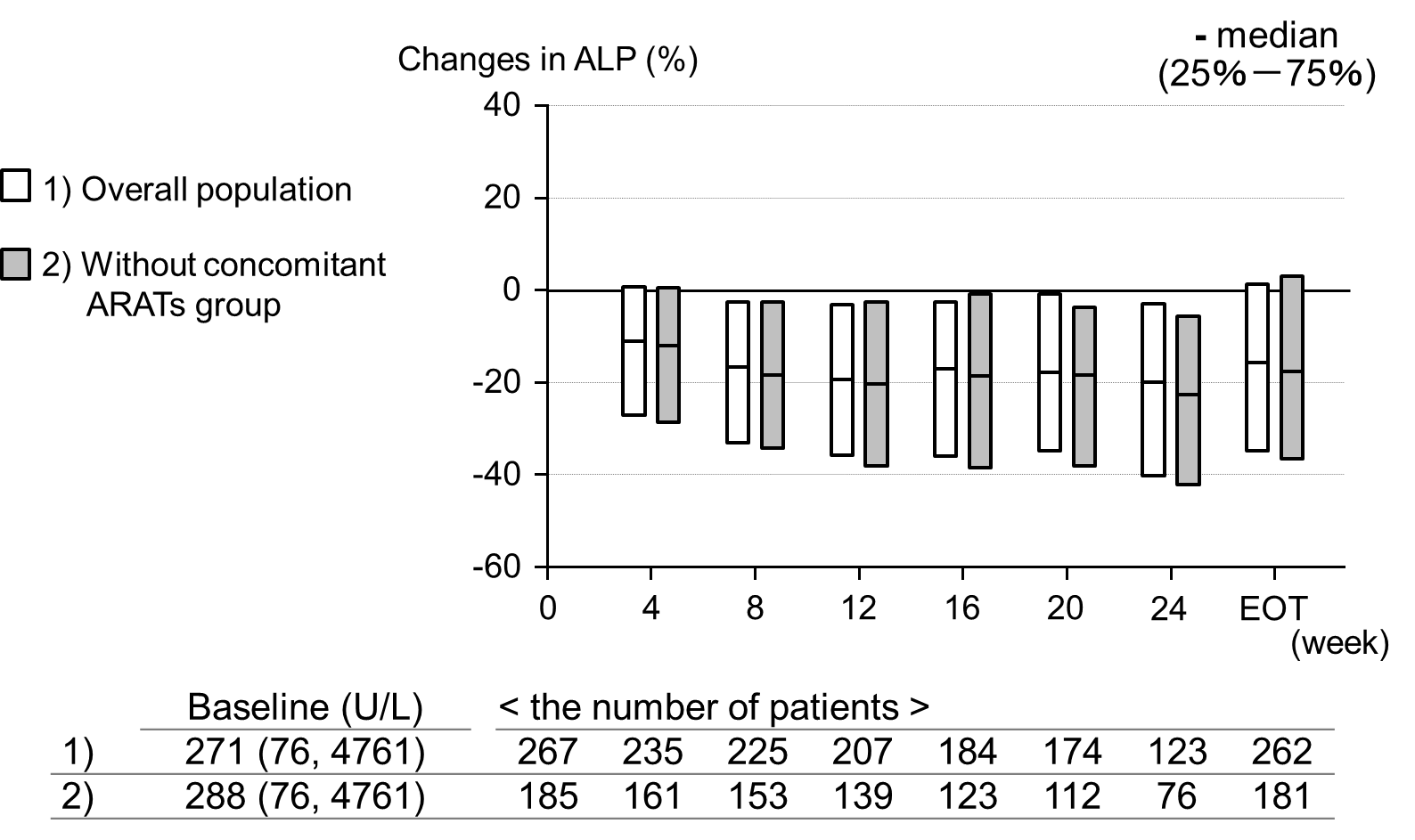
**

**b)**

**
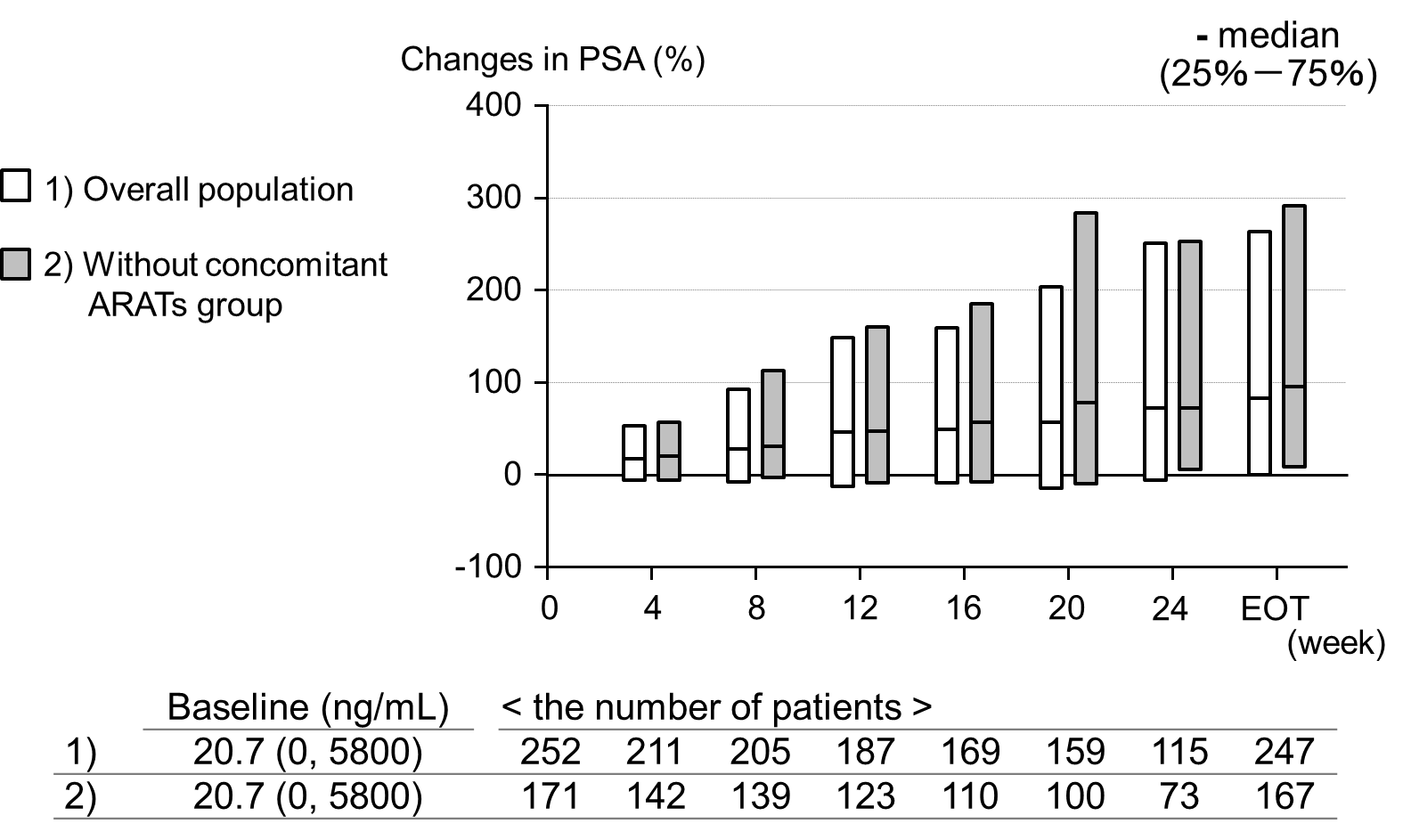
**

Each bar describes median (25% quartile -75% quartile).

ARAT = androgen-receptor axis-targeted agent; EOT=end of treatment
